# Supplementary material for: The molecular basis of phosphite and hypophosphite recognition by ABC-transporters
Source: Nat Commun. 2017 Nov 23;8:1746. doi: 10.1038/s41467-017-01226-8 (PMC5700983; doi:10.1038/s41467-017-01226-8)
Supplement: Supplementary file 1 — Supplementary Information [file 41467_2017_1226_MOESM1_ESM.pdf]

Supplementary Information

Supplementary Figures

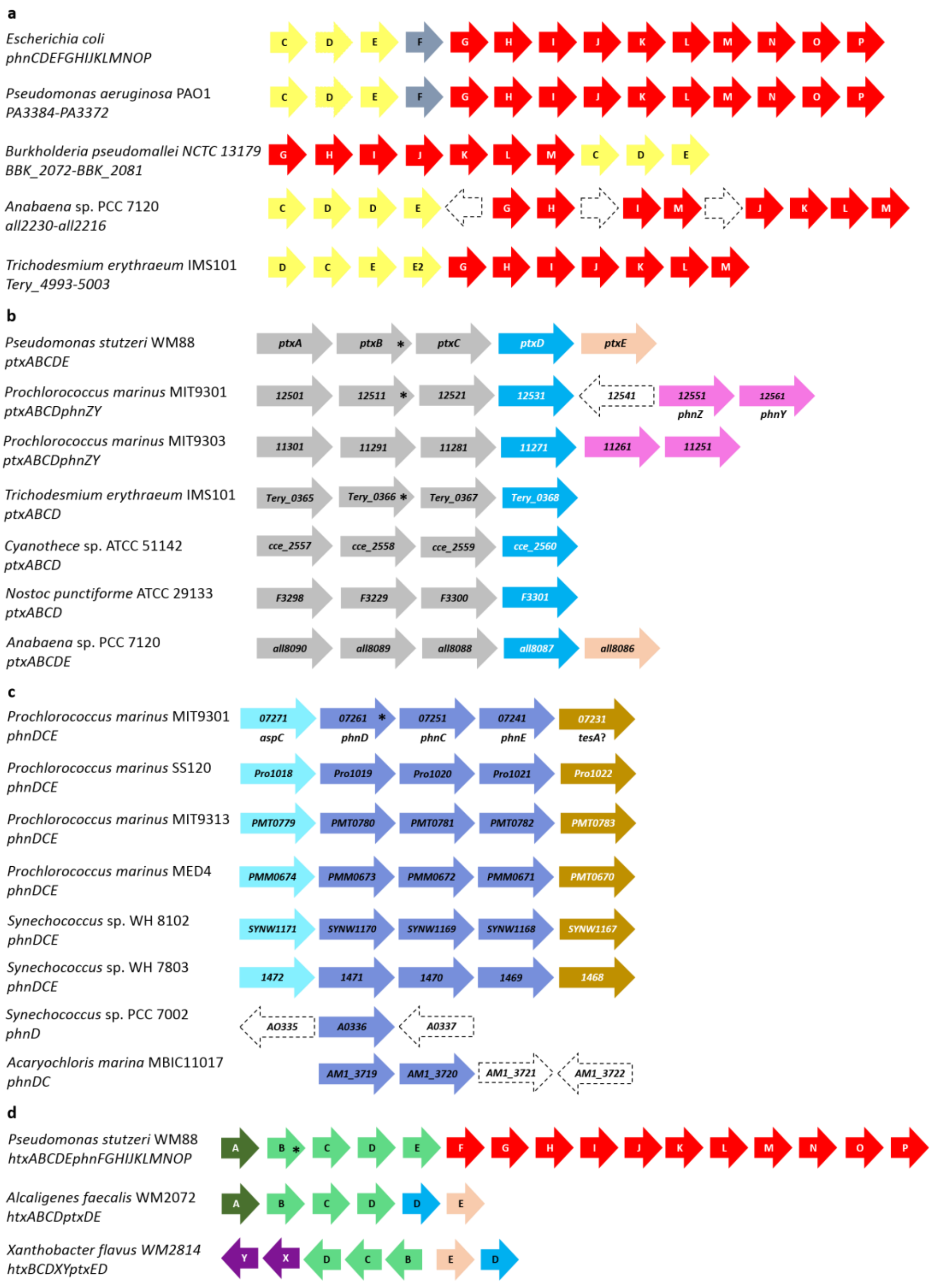

Supplementary Figure 1. Examples of genetic organisation of *phn* (C-P lyase), *ptx* and *htx* operons. **(a)** C-P lyase *phn* operons. *phnCDE* (yellow) = ABC-transporter; *phnF* (teal) =

regulator; *phnG-P* (red) = C-P-lyase catalytic and accessory proteins. **(b)** *ptx* operons. *ptxABC* (grey) = ABC-transporter; *ptxD* (cyan) = phosphite dehydrogenase; *ptxE* (peach) = LysR family transcriptional regulator; *phnZY* (pink) = *phnZ* encodes a putative Fe(II)-dependent enzyme of the histidine-aspartate motif hydrolase family and *phnY* encodes a 2-oxgluterate/Fe(II)-dependent dioxygenase[1]. The sequential activities of PhnZ and PhnY enzymes isolated from a planktonic fosmid library cleave the CP bond of 2AEPn *in vitro*[2], however the *Prochlorococcus* enzymes likely have a different unknown substrate as MIT9301 and MIT9303 cannot grow on 2AEPn, nor do the enzymes allow 2AEPn utilisation when produced in *E. coli*[3]. **(c)** *phnDCE* operons of marine cyanobacteria. *phnDCE* (lilac) = ABC-transporter; *aspC* (pale blue) = conserved putative aspartate aminotransferase; *tesA* (gold) = conserved putative lysophospholipase/(aryl)esterase. **(d)** *htx* operons. *htxA* (dark green) = hypophosphite/2-oxogluterate dioxygenase; *htxBCDE* (light green) = ABC-transporter; *htxXY* (purple) = homologues of the β- and α-subunits of soluble molybdopterin-dependent NAD:formate oxidoreductase[4]. Homologues of *phnFGHIJKLMNOP* (red) encoding C-P-lyase catalytic and accessory proteins and *ptxD* (blue, phosphite dehydrogenase) and *ptxE* (peach, putative transcriptional regulator) are found in some *htx* gene clusters. In all panels, dotted genes encode hypothetical or non-conserved proteins. The proteins characterised in this study are indicated by asterisks (*). Genes are not sized to scale for clarity.

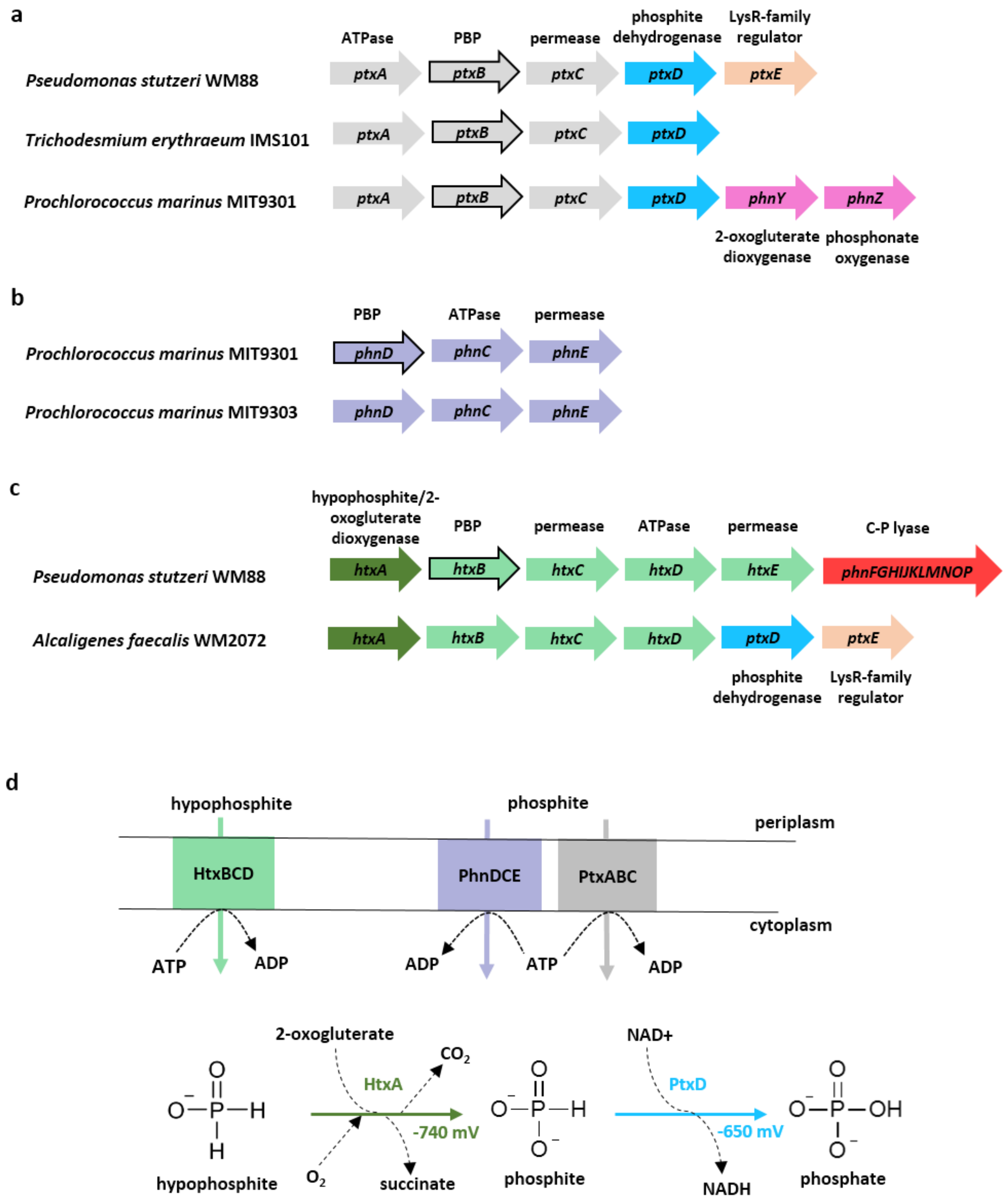

Supplementary Figure 2. Overview of phosphite and hypophosphite transporters described in this study. **(a-c)** Examples of the genetic arrangement of phosphite and hypophosphite utilisation operons. The periplasmic binding protein (PBP) components studied in this work are outlined in black. **(a)** PtxD-linked PtxABC transporters from *Pseudomonas stutzeri* WM88,

Trichodesmim erythraeum IMS101 and *Prochlorococcus marinus* MIT9301. **(b)** The PhnDCE transporter present in *Prochlorococcus* ecotypes. **(c)** HtxA-linked HtcBCD(E) transporters from *Pseudomonas stutzeri* WM88 and *Alcaligenes faecalis* WM2072. **(d)** Schematic illustration of phosphite and hypophosphite uptake and oxidation. Following uptake across the cytoplasmic membrane by the ABC-transporters, hypophosphite is converted to phosphite by the hypophosphite:2-oxogluterate dioxygenase, HtxA, and phosphite is oxidised to phosphate by the phosphite dehydrogenase, PtxD. The midpoint redox potentials at pH 7.0 of the hypophosphite/phosphite and phosphite/phosphate couples are shown. Phosphite is shown in di-anionic form (HPO_3^{2-}) and phosphate as hydrogen phosphate (HPO_4^{2-}).

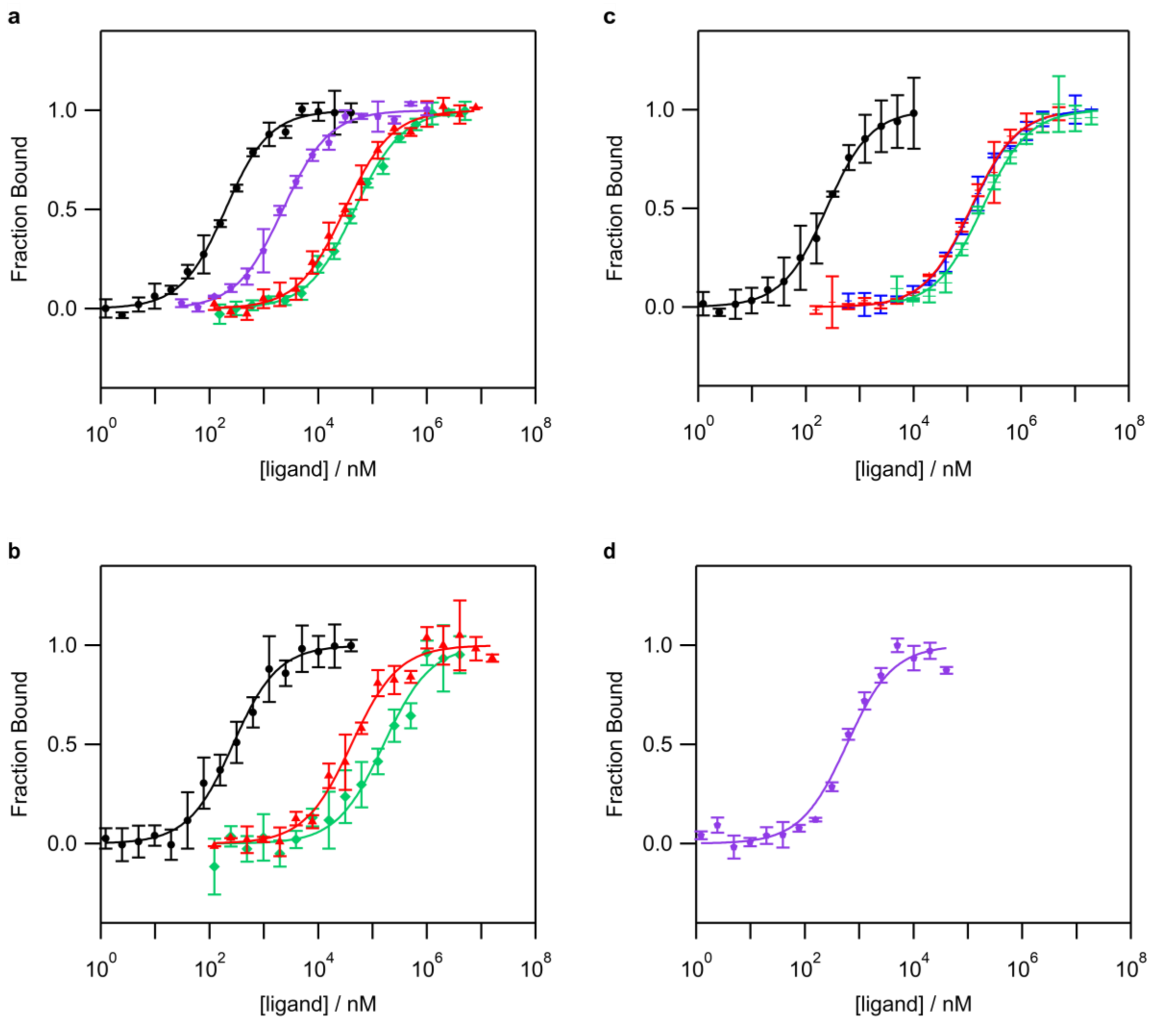

Supplementary Figure 3. Thermophoresis determined binding affinities of Te_PtxB (a), Ps_PtxB (b), Pm_PhnD (c) and HtxB (d) for phosphorus ligands. Proteins were labelled with RED-tris-NTA dye (50 nM final concentration) and MST performed in 50 mM HEPES pH 7.4, 250 mM NaCl, 0.05 % Tween-20 with proteins subjected to 22 seconds of thermophoresis. As appropriate, labelled proteins were mixed with serial dilutions of phosphite (black), methylphosphonate (red), ethylphosphonate (blue), hypophosphite (purple) and phosphate (green). Error bars indicate the standard deviation from the mean of three independent titrations.

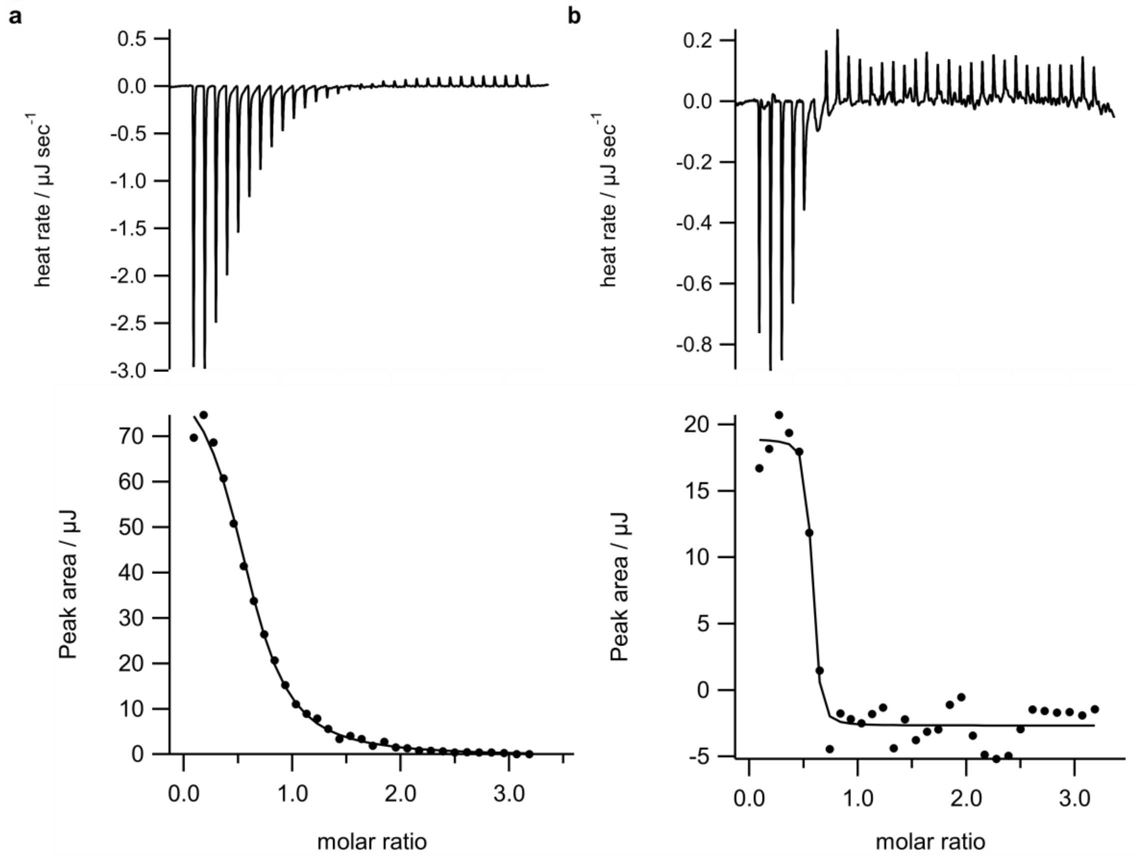

Supplementary Figure 4. Isothermal titration calorimetry produces dissociation constants in the same order of magnitude as Microscale Thermophoresis. 200 µM Te_PtxB in 50 mM HEPES pH 7.4, 250 mM NaCl, 0.05 % Tween-20 was titrated with **(a)** 2 mM phosphate (K_d = 15.9 ± 0.36 µM) and **(b)** 2 mM phosphite (K_d = 0.289 ± 0.064 µM).

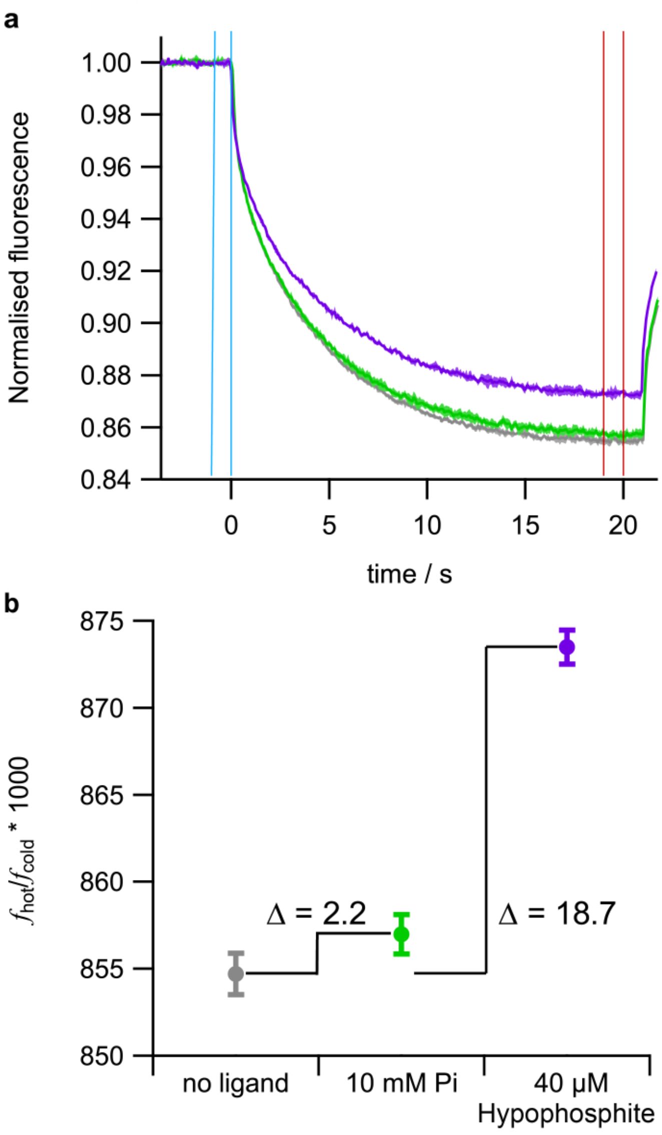

Supplementary Figure 5. HtxB does not bind phosphate. (a) Normalised thermophoresis traces of HtxB subjected to 22 seconds of thermophoresis in 50 mM HEPES pH 7.4, 250 mM NaCl, 0.05 % Tween-20 (grey), and mixed with either 10 mM phosphate (green) or 40 µM hypophosphite (purple). Lines are mean fluorescence of 4 independent measurements (standard deviation shown by shading). Blue and red lines indicate the time periods where the average of f_{hot}/f_{cold} ratio is calculated. **(b)** Category plot highlighting the difference in thermophoresis between HtxB with 10 mM phosphate and 40 µM hypophosphite. The small difference between no ligand and 10 mM phosphate is below the signal to noise ratio (S/N)

of the Monolith NT.115 machine and no binding can be concluded. The large difference shown in the presence of 40 µM hypophosphite is above the S/N (>5 units) and binding can be concluded. Error bars indicate the standard deviation from the mean of four independent experiments.

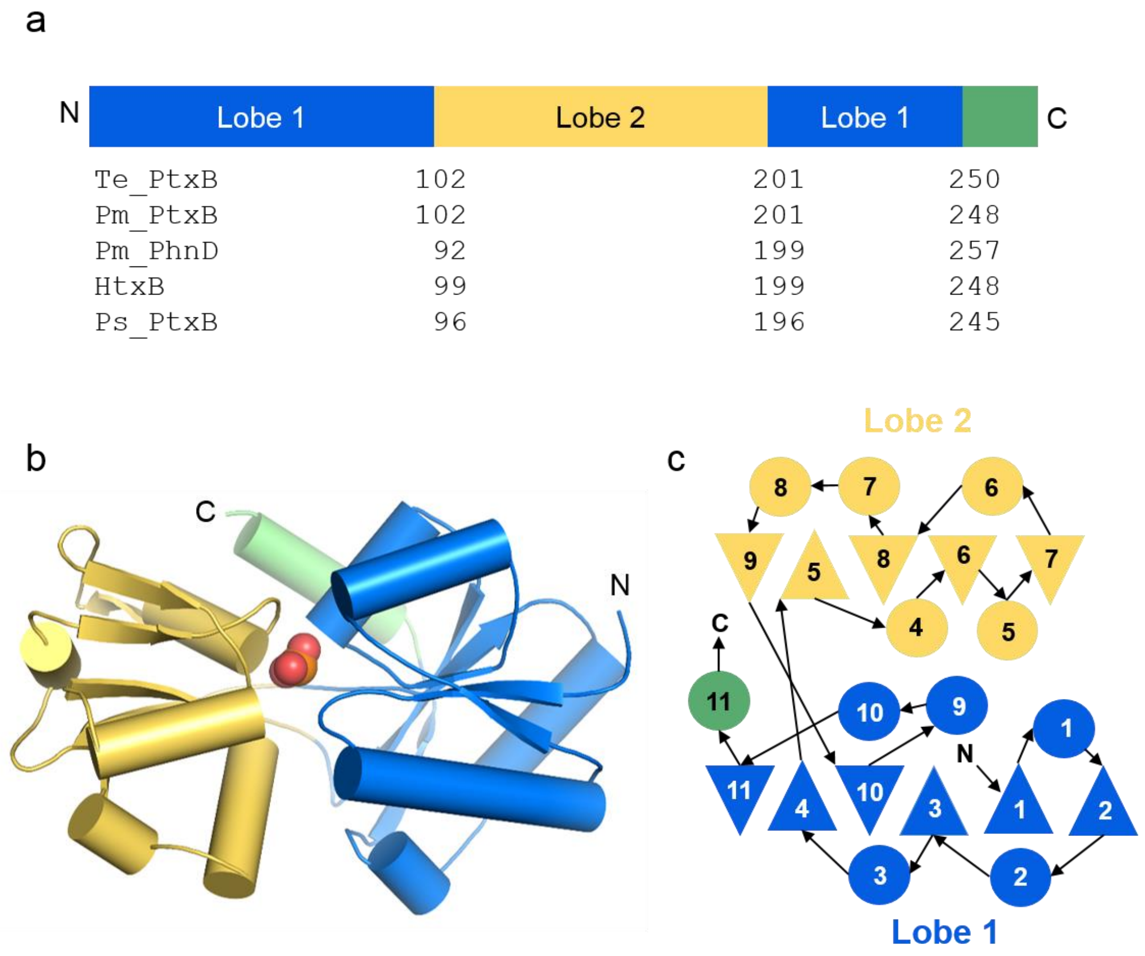

Supplementary Figure 6. The domain organisation and overall topology of Te_PtxB, Pm_PtxB, Pm_PhnD, HtxB and Ps_PtxB. (a) Block diagram representation of the primary structure of the five proteins. Lobe 1 (blue and green) is the larger of the two domains and formed from the two termini of the protein, whilst lobe 2 (yellow) is formed from a ~100 amino acid stretch in the middle of each polypeptide. The C-terminal helix is shown in green. The amino acids that determine the border of each domain are numbered and shown for each protein below the schematic. **(b)** A cartoon representation of the protein fold modelled on Te_PtxB and **(c)** a topology schematic describing the connectivity of the secondary structure elements, coloured as in **(a)**; α-helices are depicted as circles and β-sheets as triangles.

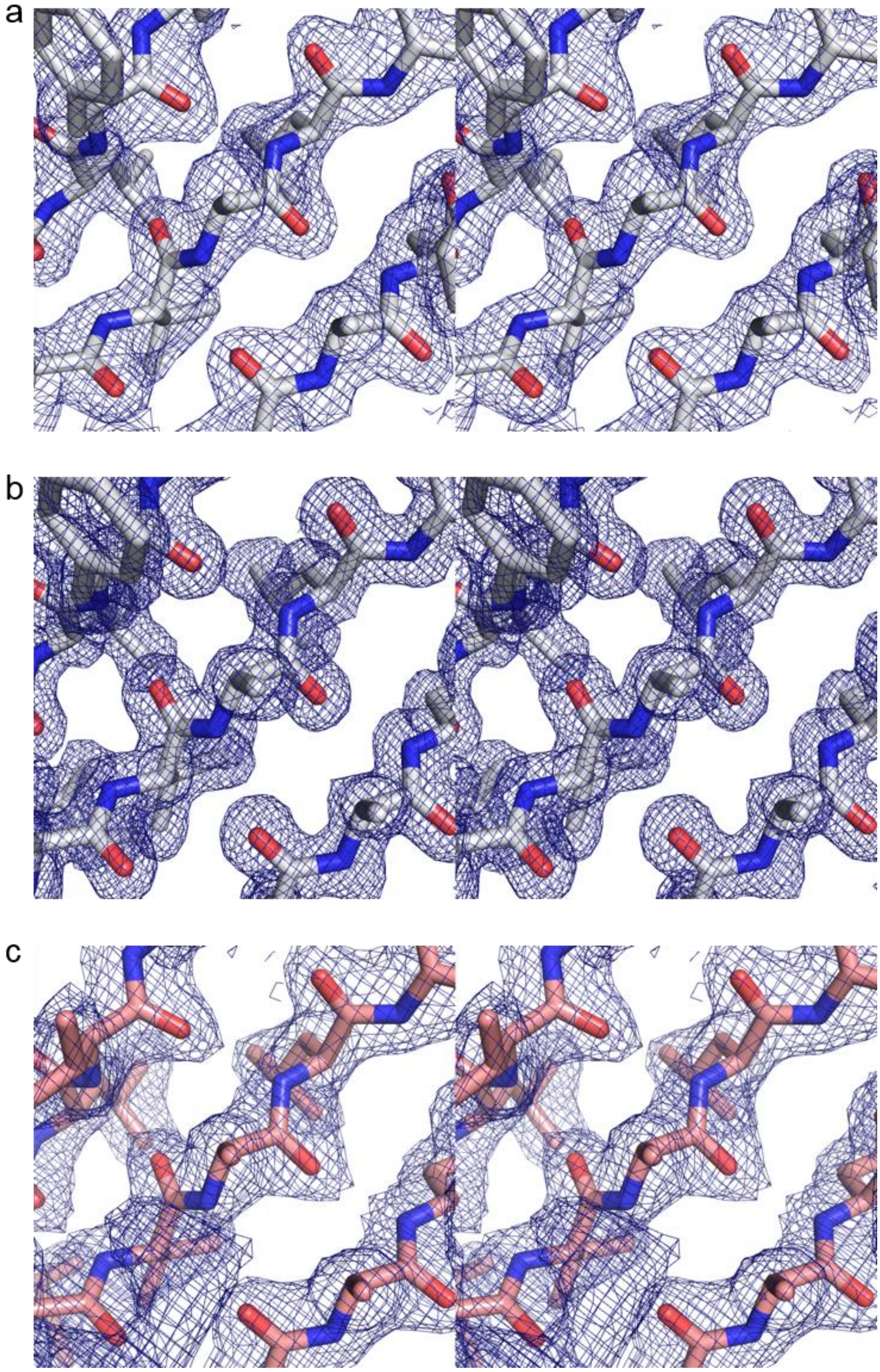

Supplementary Figure 7. **Stereo images of a portion of the 2Fo-Fc electron density map part 1.** A section of the map (blue mesh, contoured at 1.0 σ) surrounding an area of beta-sheet is shown from the Te_PtxB/phosphite **(a)**, Te_PtxB/MPn **(b)** and Pm_PtxB/phosphite **(c)** complexes.

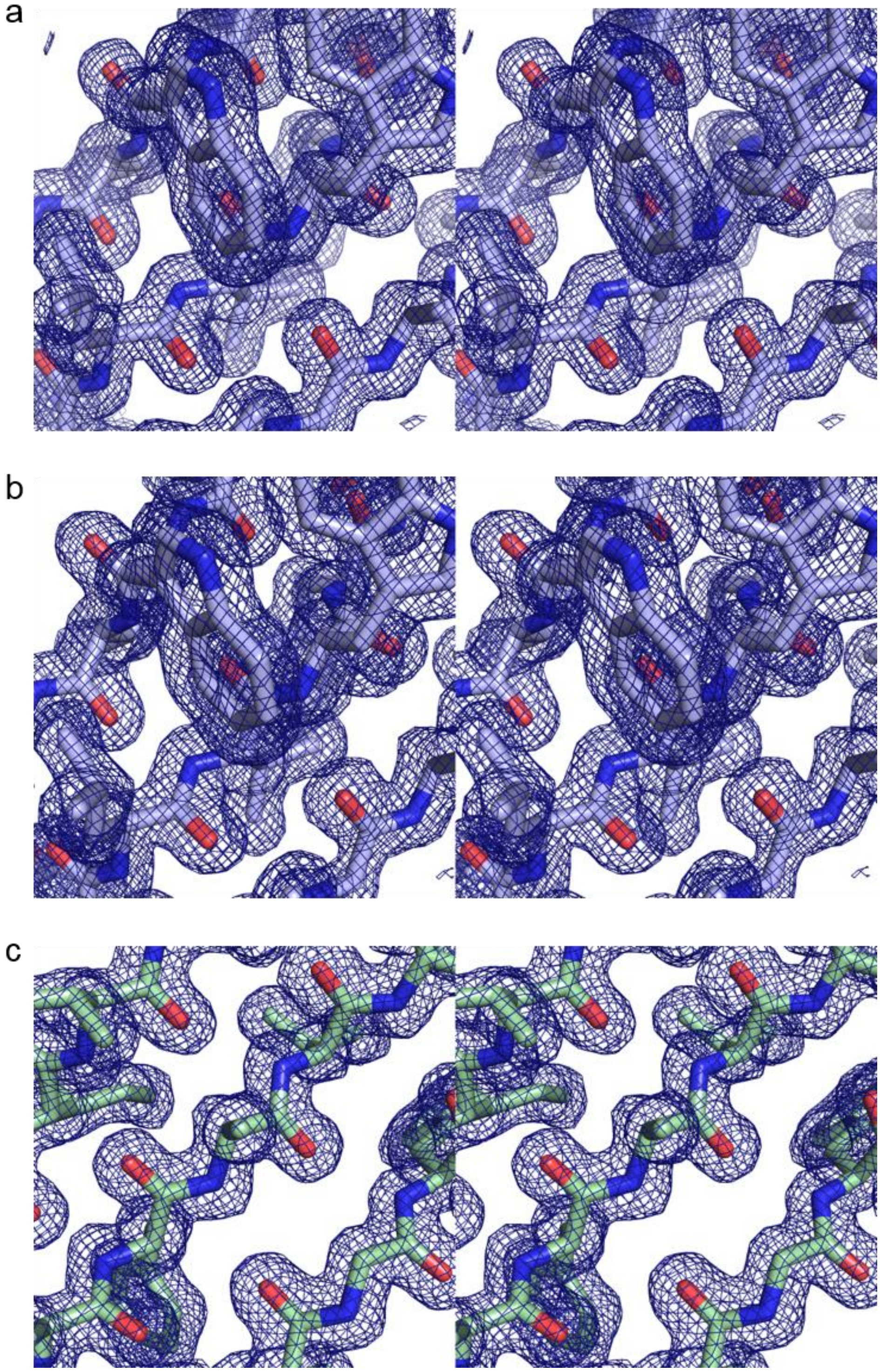

Supplementary Figure 8. Stereo images of a portion of the 2Fo-Fc electron density map part 2. A section of the map (blue mesh, contoured at 1.0 σ) surrounding an area of beta-sheet is shown from the Pm_PhnD/phosphite **(a)**, Pm_PhnD/MPn **(b)** and HtxB/hypophosphite **(c)** complexes.

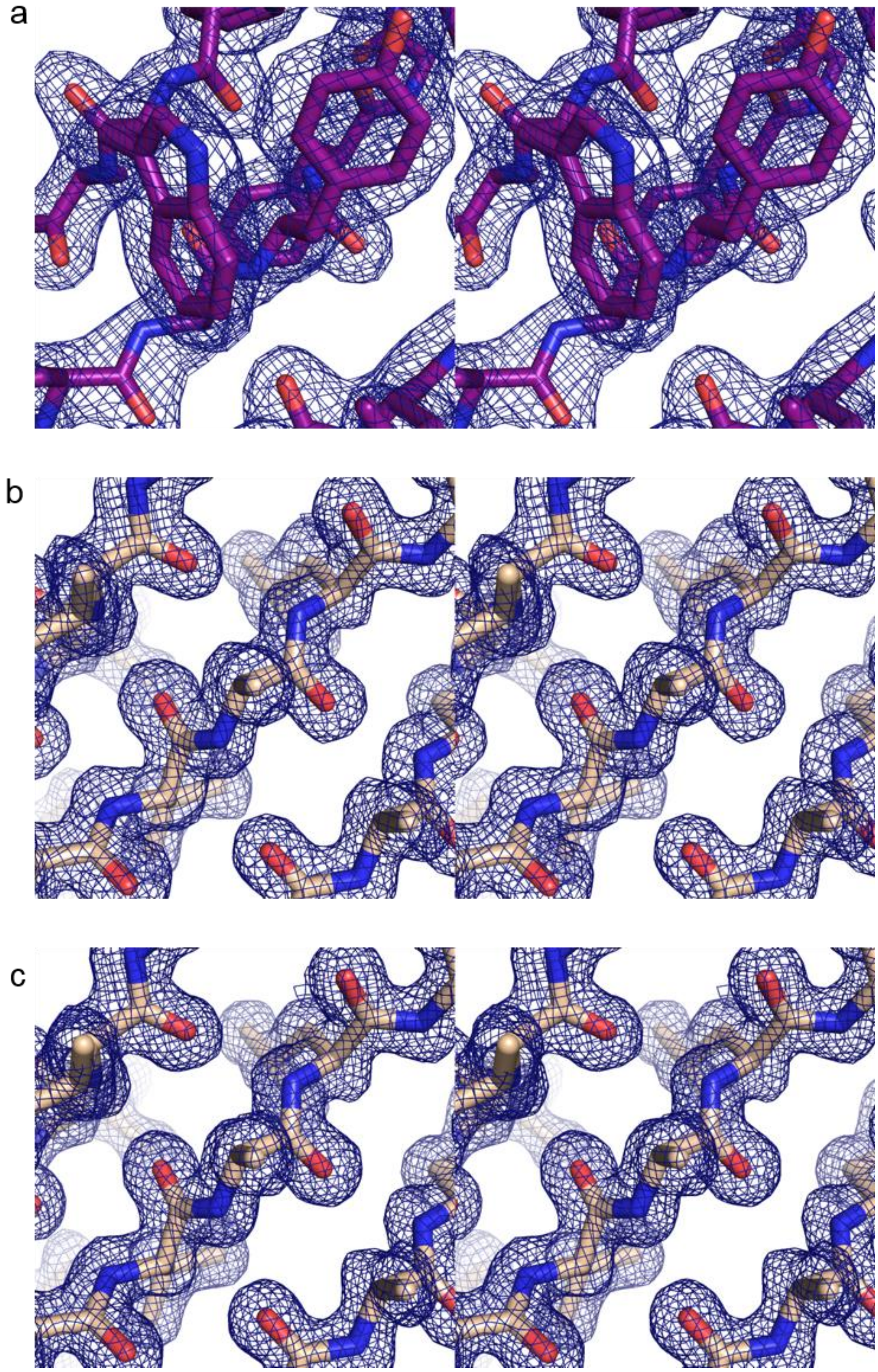

Supplementary Figure 9. Stereo images of a portion of the 2Fo-Fc electron density map part 3. A section of the map (blue mesh, contoured at 1.0 σ) surrounding an area of beta-sheet is shown from the apo Ps_PtxB **(a)**, Ps_PtxB/phosphite **(b)** and Ps_PtxB/MPn **(c)** complexes.

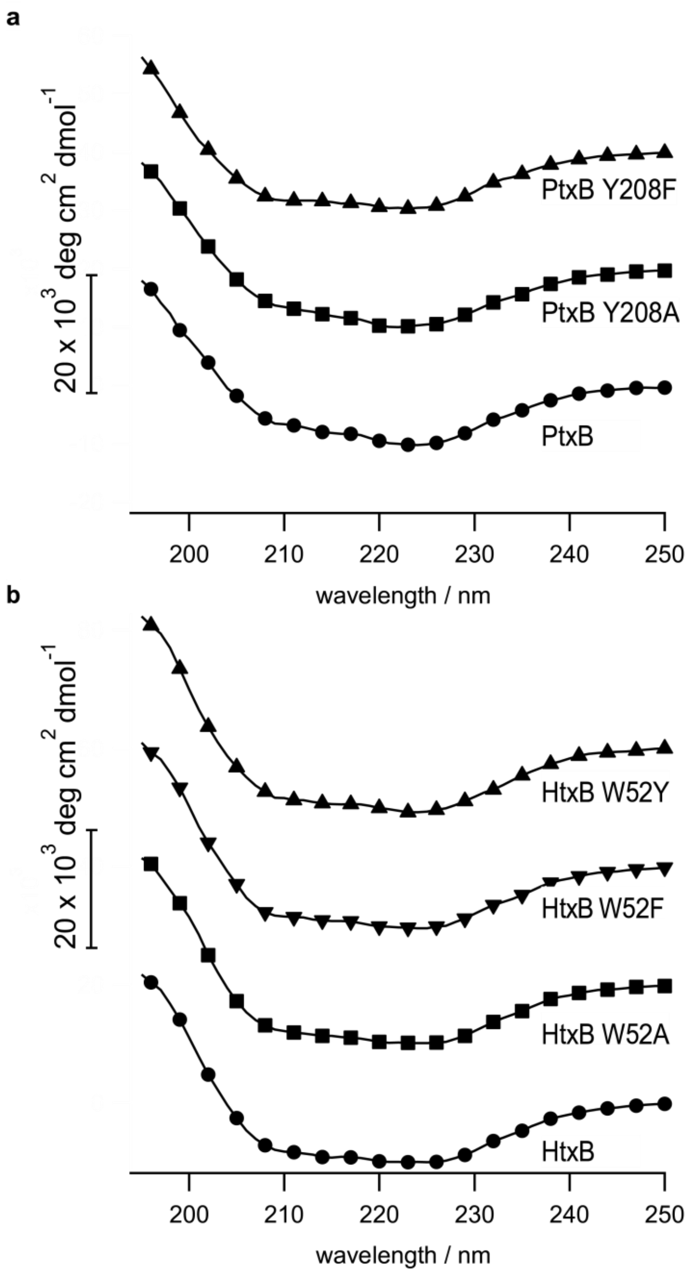

Supplementary Figure 10. Mutations in the binding pocket of Te_PtxB (a) and HtxB (b) do not change the secondary structure of the protein. CD spectra (mean residue ellipticity) measured at 25 °C in 5 mM sodium phosphate buffer, pH 7.4.

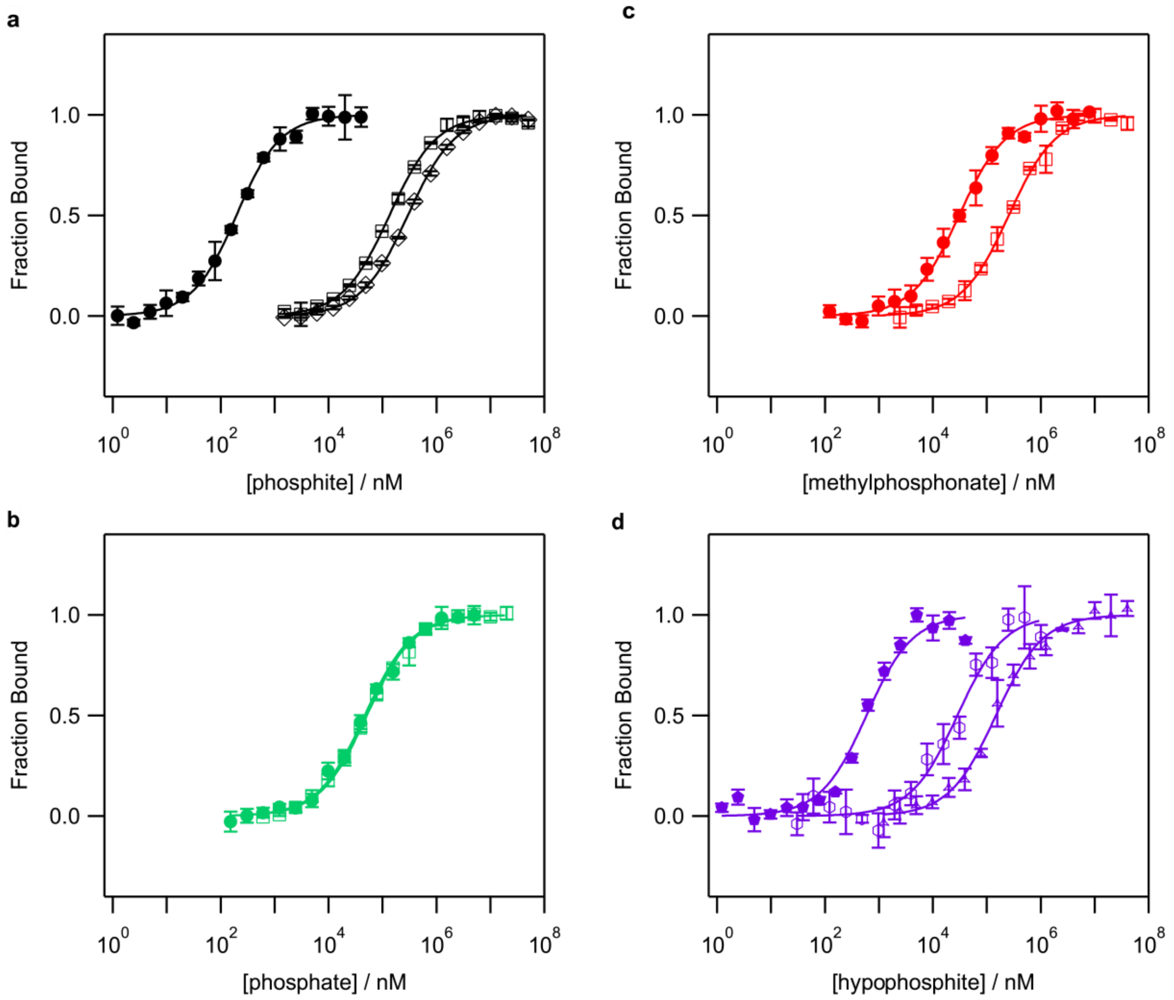

Supplementary Figure 11. Mutations in the binding pocket of Te_PtxB and HtxB reduce the affinity for phosphite and hypophosphite respectively. **(a)** Te_PtxB (●), Y208A (☐) and Y208F (◇) titrated with phosphite. **(b)** Te_PtxB (●) and Y208A (☐) titrated with phosphate. **(c)** Te_PtxB (●) and Y208A (☐) titrated with methylphosphonate. **(d)** HtxB (⬟), W52F (⬡) and W52Y (⅄) titrated with hypophosphite. Proteins were labelled with RED-tris-NTA dye (50 nM final concentration) and MST performed in 50 mM HEPES pH 7.4, 250 mM NaCl, 0.05 % Tween-20, with proteins subjected to 22 seconds of thermophoresis. Error bars indicate the standard deviation from the mean of three independent titrations.

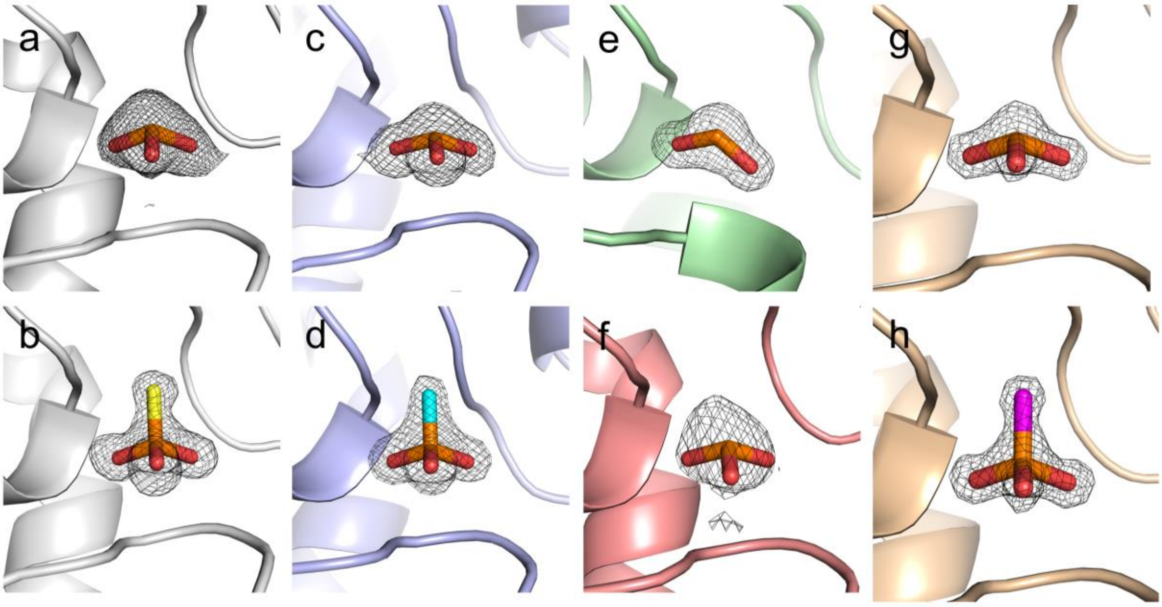

Supplementary Figure 12. **Omit maps.** Overall omit maps (grey mesh, contoured at 3σ) were generated using SFCHECK[5] (sticks, O=red, P=orange; MPn methyl group=yellow, cyan or magenta) for Te_PtxB in complex with phosphite **(a)** and MPn **(b)**; Pm_PhnD with phosphite **(c)** and MPn **(d)**; HtxB with hypophosphite **(e)**; Pm_PtxB with phosphite **(f)**; and Ps_PtxB with phosphite **(g)** and MPn **(h)**.

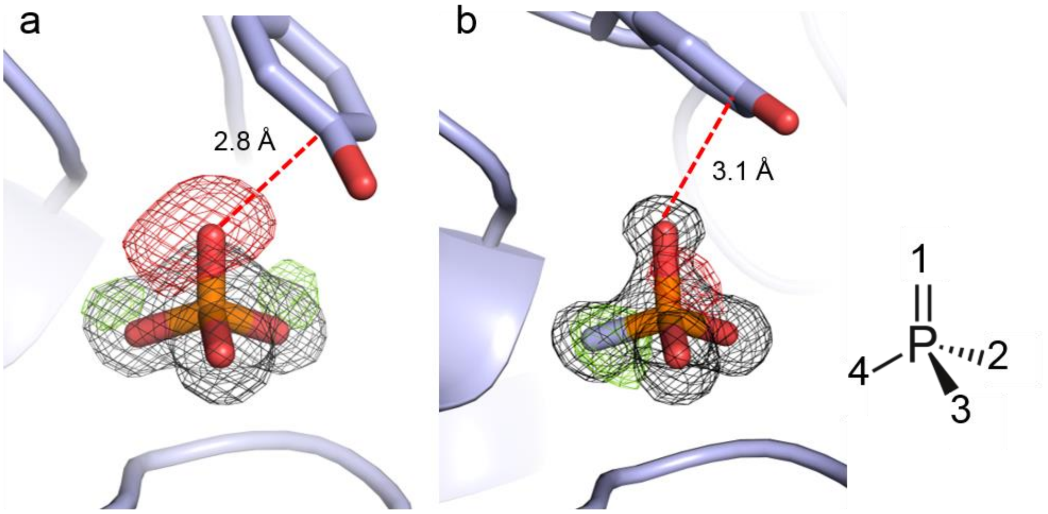

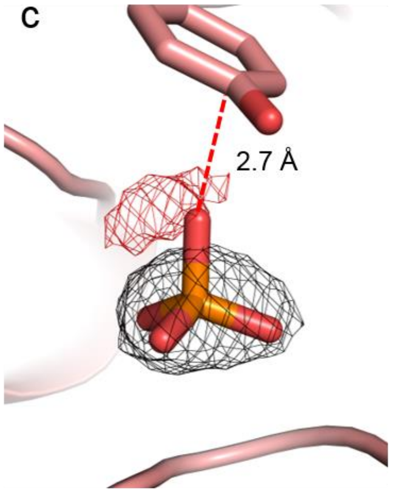

d

	Bfactors (Å²)		
	a	b	c
Structure	Pm_PhnD/Pst	Pm_PhnD/MPn	Pm_PtxB/Pst (PO_4 buffer)
Validation	PO_4 modelled	MPn rotated	PO_4 modelled
P	17.85	8.51	24.1
1	30.54	11.20	97.36
2	18.64	8.5	24.74
3	18.03	8.85	19.4
4	19.51	6.25	18.53

Supplementary Figure 13. Ligand validation. An example of some of the post-refinement 2Fo-Fc maps (grey) and Fo-Fc difference maps (green and red) generated during validation of the ligand binding. In **(a)** a phosphate moiety has been modelled and refined into the ligand density in the Pm_PhnD complex with phosphite, producing a large negative difference peak on the R1 oxygen position. In **(b)** the MPn in the Pm_PhnD/MPn complex is rotated. Positive and negative difference features identify the correct orientation of the ligand. **(c)** Even though the Pm_PtxB protein was crystallised in phosphate buffer, the ligand density cannot be explained by a phosphate moiety, signified by the large negative difference peak at the R1 position. **(d)** A table of B-factors accompanies the maps.

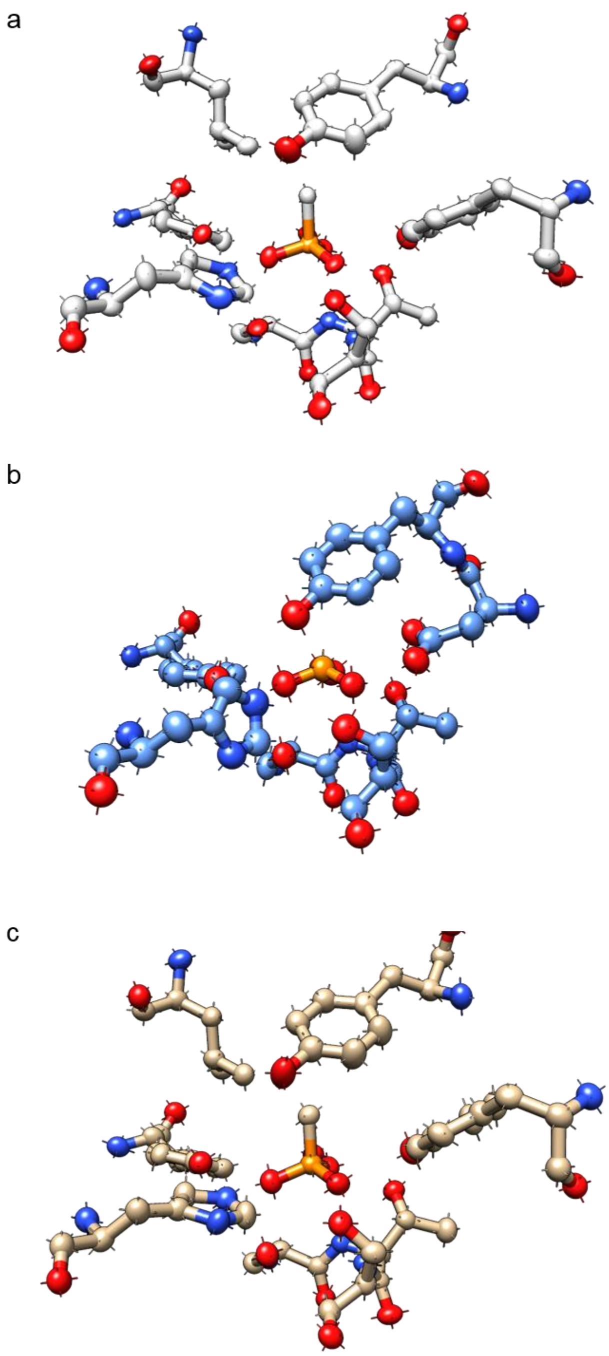

Supplementary Figure 14. Thermal ellipsoids. Thermal ellipsoids were calculated for atoms surrounding the binding pocket and for the ligand from anisotropic B-factors in Chimera[6] and are represented with principal axes shown as lines. Ellipsoids are coloured in atom colours (O=red, N=blue and P=orange), with carbon atoms coloured grey for Te_PtxB/MPn **(a)**, blue for Pm_PhnD/phosphite **(b)** and beige for Ps_PtxB/MPn **(c)**. The resolution of these structures is 1.41 Å, 1.46 Å and 1.37 Å, respectively.

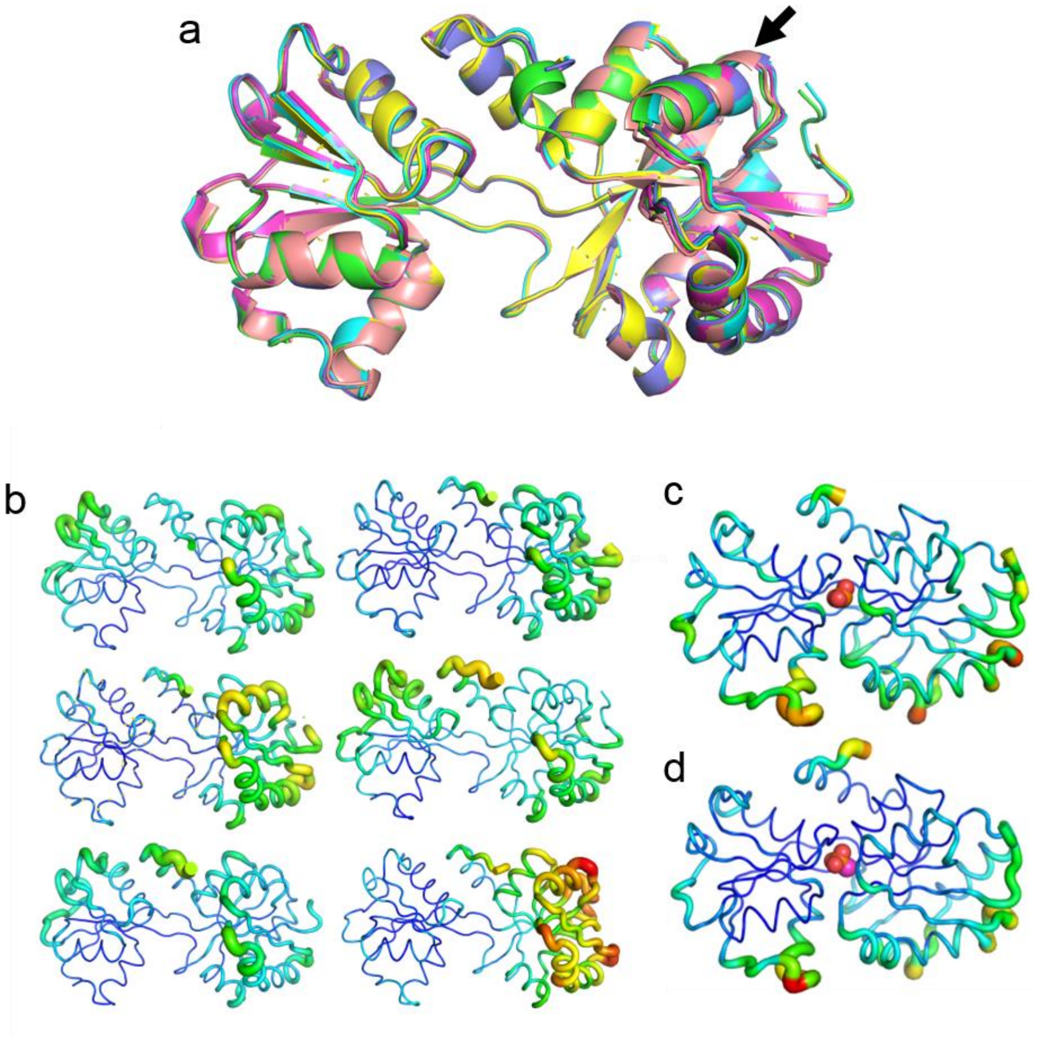

Supplementary Figure 15. Flexibility within the Ps_PtxB apo protein. (a) The six molecules within the asymmetric unit of the apo Ps_PtxB crystal structure are shown as cartoon ribbons and superimposed. The only notable difference is in the conformation of helix 2 (black arrow), which differs in position by a maximum of 2 Å across the six molecules. **(b)** Displaying the six chains as B-factor putty shows how the B-factors in lobe 1 of each molecule are generally higher (green/red/yellow, fat tubes) that lobe 2 (blue, thin tubes), signifying a degree of flexibility within the crystal lattice. **(c-d)** The same representation of the Ps_PtxB complexes with phosphite **(c)** and methylphoshonate **(d)** shows how the B-factors in the core of the binding pocket are reduced (blue, thin tubes) whilst the periphery of each lobe has more flexible regions that are equivalent in both complexes.

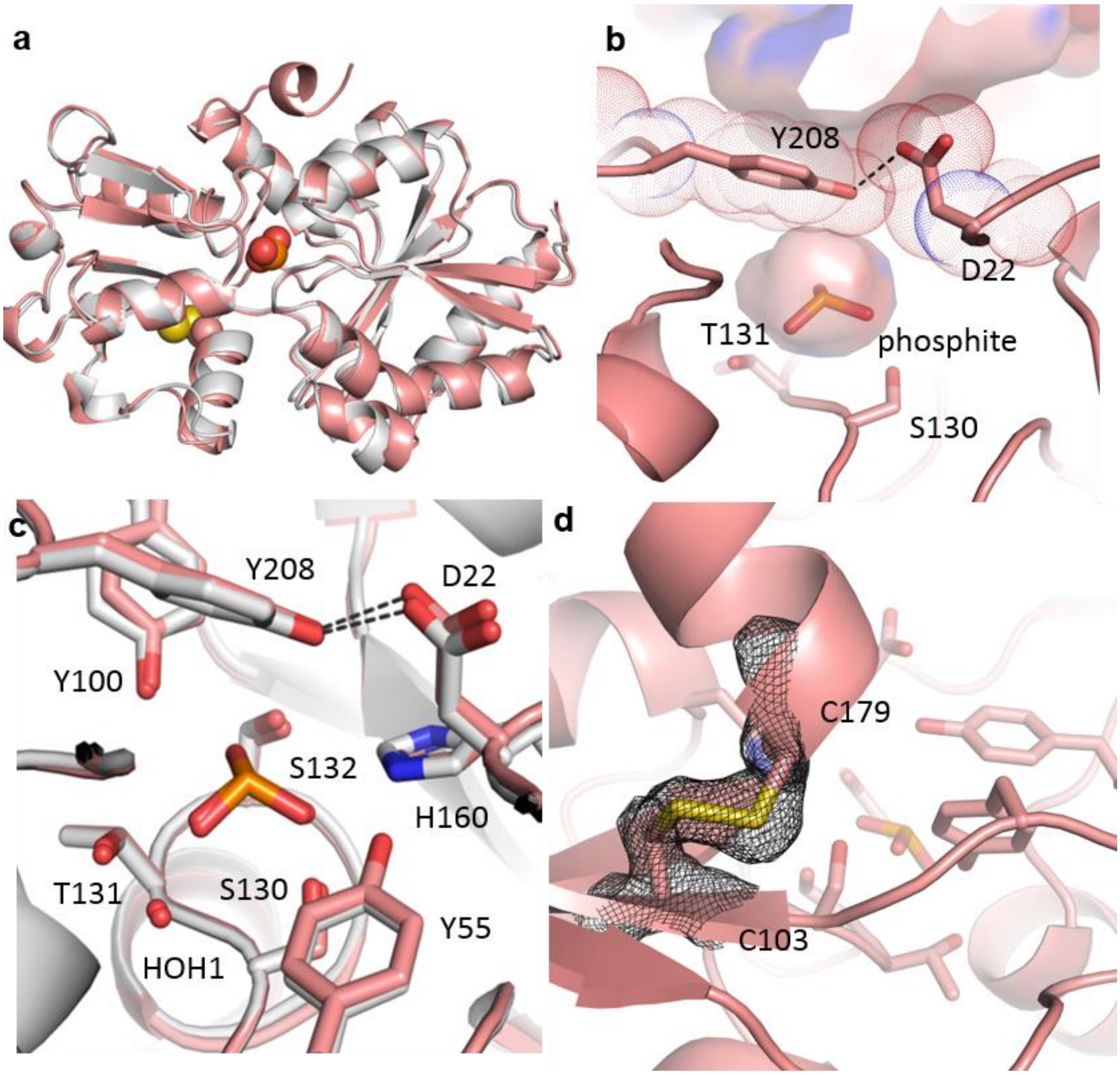

Supplementary Figure 16. The structure of the *Prochlorococcus marinus* MIT9301 PtxB (Pm_PtxB) in complex with phosphite and its structural similarity to Te_PtxB. (a) A comparison of the overall fold of Te_PtxB (grey) and Pm_PtxB (pink). The protein backbones are shown as cartoons with the phosphite ligand and disulphide bond between C103 and C179 shown as spheres and coloured by atom (S = yellow). **(b)** The Y/D capping residues in Pm_PtxB (sticks and dotted van der Waals surface) separate the internal cavity of the binding pocket from the solvent (partially transparent surface representations); hydrogen bonds are shown as black dashes. **(c)** A detailed view of the binding pocket of both structures showing that the architecture surrounding the phosphite is essentially identical and with residue numbers (the

same in both proteins) highlighted. **(d)** The final refined 2Fo-Fc map surrounding the disulphide bond in Pm_PtxB is contoured at 1.2σ and drawn as a grey mesh.

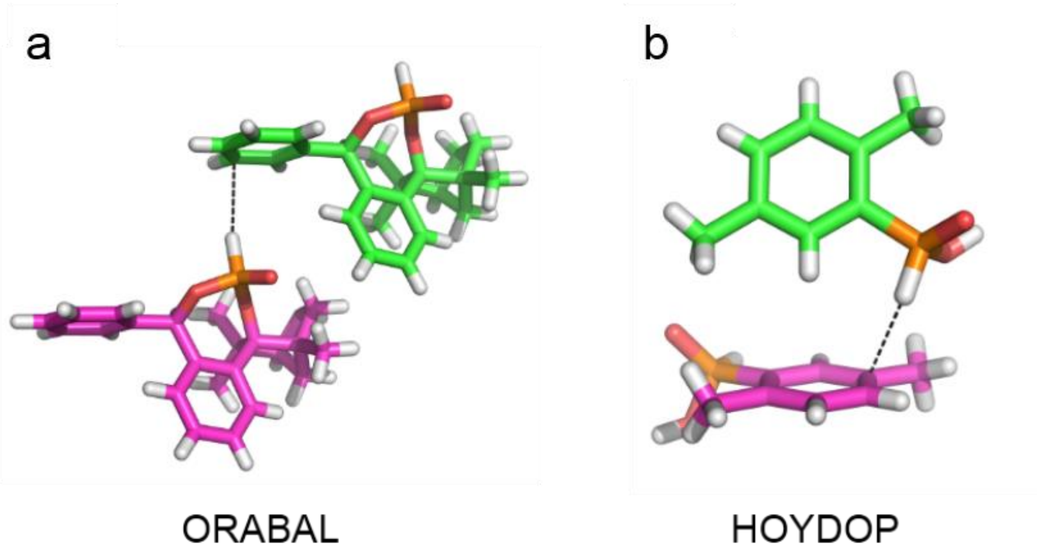

Supplementary Figure 17. Examples of P-H…π interactions in small molecule crystal structures in the Cambridge Structural Database. Examples of two of the molecules from Supplementary Table 2, highlighting the P-H…π interaction (black dashed line). (a) ORABAL, 1',3',3'-Trimethyl-5-phenyl-5H-spiro[2,4,3-benzodioxaphosphepine-1,2' bicyclo[2.2.1] heptane] 3-oxide. (b) HOYDOP, (2,5-Dimethylphenyl)phosphinic acid) Neighbouring molecules are drawn as sticks and coloured pink and green, with other atoms in atom colours (H=white, O=red, P=orange).

Supplementary Tables

Protein	Ligand	Binding pocket volume (Å³)	Difference in binding pocket volume (%)		
			MPn vs phosphite	vs Pm_PhnD/ phosphite complex	vs Te_PtxB/ phopshite complex
Te_PtxB	Phosphite	92	↑ 16		
	MPn	108			
Ps_PtxB	Phosphite	98	↑ 16		
	MPn	115			
Pm_PhnD	Phosphite	86	↑ 8		
	MPn	93			
Pm_PtxB	Phosphite	116		↑ 29	↑ 23
HtxB	Hypophosphite	68		↓ 23	↓ 36

Supplementary Table 1. Volume of the binding pockets when occupied by different ligands. The volume of the internal cavity that forms the binding pocket were calculated via the CASTp server[7] using a probe radius of 1.4 Å and analysed in Chimera[6]. Volumes are given in $Å^3$ for each structure. The percentage increase in binding pocket volume when MPn rather than phosphite is bound is given where applicable. Where there was more than one protein molecule in the asymmetric unit the mean volume was recorded.

CSD Refcode	Small molecule	P-H…π plane distance (Å)	P-H…π angle (°)	Ref.
HOKNIG	Methylenebis(phenylphosphine) dioxide	2.64	155	8
HOKNUS	Phenyl((phenylphosphino)methyl) phosphine oxide	2.73	120	8
CEPYUS01	Tritylphosphinic acid	2.62	125	9
CEPZAZ	Tritylphosphine oxide	2.76	160	10
GUJHUP	((R,R),S)-O,O'-Dibenzoyltartaric acid t-butylphenylphosphine oxide	2.45	130	11
HOYDOP	(2,5-Dimethylphenyl)phosphinic acid	2.59	155	12
MIYQAO	Diphenylphosphine oxide	2.00	170	13
NAPNUN	3-(4-Fluorobenzyl)-2-hydro-1-methyl-5,6-benzo-1,3,2-diazaphosphorin-4-on-2-oxide	2.70	135	14
OQUZIK*	1',3',3'-Trimethyl-5,5-bis(trifluoromethyl)-5H-spiro[2,4,3-benzodioxaphosphepine-1,2'-bicyclo[2.2.1]heptane] 3-oxide	2.07	180	15
ORABAL	1',3',3'-Trimethyl-5-phenyl-5H-spiro[2,4,3-benzodioxaphosphepine-1,2'-bicyclo[2.2.1]heptane] 3-oxide	2.71	140	15
OZIVID	5,9-Diphenyl-5,9-bis(trifluoromethyl)-5,9-dihydrodibenzo[e,g][1,3,2]dioxaphosphonine 7-oxide	2.27	130	15
RAFBOP	Bis(2-Hydroxynaphthyl)methane 2-oxo-5,5-dimethyl-1,3,2λ5-dioxaphosphorinane	2.66	165	16

Supplementary Table 2. Occurrence of P-H…π interactions in the Cambridge Structural Database (CSD). The CSD was searched for P-H…π interactions within small molecule crystal structures using Conquest to identify hydrogen atoms attached to a phosphorus atom that are within van der Waals radii of the plane of a π-system. A total of 12 structures were identified. In each case the P-H…π interaction was intermolecular and made between molecules within the asymmetric unit of the crystal. The table lists the molecules identified from the database by their Refcode, chemical name and the P-H…π interaction distance and angle. The asterisk (*) indicates that the P-H bond lies almost parallel to the plane of the aromatic group, but with the H atom within the van der Waals radii of the aromatic system.

Protein and organism	Abbreviation	KEGG Entry	NCBI Accession	UniProtKB
**PtxB**				
Pseudomonas stutzeri WM88	Pstut_PtxB	N/A	AAC71707	O69052
Klebsiella pneumonia MGH 78578	Kleb_PtxB	KPN_pKPN3p05890	ABR80269	A6TI48
Shewanella putrefaciens 200	Shew-PtxB	Sput200_3279	ADV55672	E6XMC1
Prochlorococcus marinus MIT 9301	Pm9301_PtxB	P9301_12511	ABO17874	A3PC74
Prochlorococcus marinus MIT 9302	Pm9302_PtxB	N/A	KGF97635	N/A
Prochlorococcus marinus MIT 9303	Pm9303_PtxB	P9303_11291	ABM77878	A2C8R8
Trichodesmium erythraeum IMS101	Te_PtxB	Tery_0366	ABG49835	Q119I9
Cyanothece sp. ATCC 51142	51142_PtxB	cce_2558	ACB51906	B1WSC0
Nostoc punctiforme ATCC 29133	Nostoc_PtxB	Npun_F3299	ACC81739	B2IZW8
Anabaena sp. PCC 7120	7120_PtxB	all8089	BAB77419	Q8YK29
**PhnD**				
Prochlorococcus marinus MIT 9301	9301_PhnD	P9301_07261	ABO17349	A3PC74
Prochlorococcus marinus MIT 9303	9303_PhnD	P9303_14311	ABM78177	A2C9L7
Prochlorococcus marinus MED4	MED4_PhnD	PMM0673	CAE19132	Q7V218
Prochlorococcus marinus SS120	SS120_PhnD	Pro_1019	NP_875411	Q7VBS3
Prochlorococcus marinus MIT 9313	9313_PhnD	PMT_0780	CAE20955	Q7V7G6
Prochlorococcus marinus MIT 9515	9515_PhnD	P9515_07461	ABM71955	A2BVZ4
Prochlorococcus marinus MIT 9211	9211_PhnD	P9211_07861	ABX08717	A9BA55
Prochlorococcus marinus NATL1A	NATL1_PhnD	NATL1_07301	ABM75288	A2C1C8
Prochlorococcus marinus AS9601	AS9601_PhnD	A9601_07281	ABM70014	A2BQF3
Synechococcus sp. PCC7002	7002_PhnD	A0336	ACA98346	B1XNG8
Acaryochloris marina MBIC11017	Am_PhnD	AM1_3719	ABW28709	B0C4G5
Synechococcus sp. WH8102	WH8102_PhnD	SYNW1170	CAE07685	Q7U716
Synechococcus sp. WH7803	7803_PhnD	SynWH7803_1471	CAK23897	A5GLT2
Synechococcus sp. CC9311	CC9311_PhnD	sync_1670	ABI45911	Q0I9J8
Synechococcus sp. CC9605	CC9605_PhnD	Syncc9605_1096	ABB34853	Q3AKM9
Synechococcus sp. RCC307	RCC307_PhnD	SynRCC307_1457	CAK28360	A5GU01
**C-P lyase PhnD**				
Escherichia coli K-12 MG1655	Ec_C-P	b4105	NP_418529	P16682
Pseudomonas aeruginosa PAO1	Pa_C-P	PA3383	NP_252073	Q9HYL8
Burkholderia pseudomallei NCTC 13179	Bp_C-P	BBK_2080	AGZ27021	N/A
Anabaena sp. PCC 7120	7120_C-P1	all2228	BAB73927	Q8YUV3
Anabaena sp. PCC 7120	7120_C-P2	all2229	BAB73928	Q8YUV2
Trichodesmium erythraeum IMS101	Tery_C-P	Tery_4993	ABG53907	Q10V17
Synechococcus sp. JA-2-3B'a(2-13)	Ja-2-3B_C-P	Cyb_0160	ABD01161	Q2JPW5
Rhodopseudomonas palustris CGA009	Rpal_C-P	RPA0699	CAE26143	Q6NBX7
**HtxB**				
Pseudomonas stutzeri WM88	Pstut_HtxB	N/A	AAC71712	O69061
Delftia lacustris	Dl_HtxB	N/A	WP_047219907	N/A
Cupriavidus basilensis	Cb_HtxB	N/A	WP_059414254	N/A
Alcaligenes faecalis	Af_HtxB	N/A	AAT12776	Q5J1M1
Rhodopseudomonas sp. AAP120	Rsp_HtxB	N/A	WP_054163872	A0A0N0KHN3
Methylobacterium radiotolerans	Mr_HtxB	N/A	KZB97311	A0A154NCP7
Xanthobacter flavus	Xf_HtxB	N/A	ABG02408	A5X3G7
**Hybrid?**				
Bradyrhizobium sp. BTAi1	Bsp_Hybrid	BBta_3379	ABQ35478	A5EH34
Methylopila sp. 73B	Msp_Hybrid	N/A	WP_051459998	N/A
Lutibaculum baratangense AMV1	Lb_Hybrid	N/A	ESR24638	V4TEL6

Supplementary Table 3. Details of proteins used in sequence alignments and to generate the phylogenetic tree in Figure 5. The abbreviations used in Figure 5a are listed, as are KEGG, NCBI and UniProt identifiers, as applicable.

Name	Sequence 5'-3'	Information
Te_ptxB-F	ATGCCATATGAATATTCAAAAATCAGAGAACAAAGCTAATCC	*Nde*I site underlined
Te_ptxB-R	ATGCCTCGAGTTTATTCAATTGTTCAAAATTAAGCTCTAGCAC	*Xho*I site underlined
Te_ptxB_Y208F-F	AAACCTTTTCCTCAGTTTCCTTGGACAATGCGCTC	Altered nucleotide(s) underlined
Te_ptxB_Y208F-R	AGCGCATTGTCCAAGGAAACTGAGGAAAAGGTTTAG	Altered nucleotide(s) underlined
Te_ptxB_Y208A-F	TCTAAACCTTTTCCTCAGGCTCCTTGGACAATGCGCTCTG	Altered nucleotide(s) underlined
Te_ptxB_Y208A-R	AGAGCGCATTGTCCAAGGAGCCTGAGGAAAAGGTTTAG	Altered nucleotide(s) underlined
htxB_W52Y-F	TTTCGCCCGAGTCCTATGCGGCCATCAGCGTAG	Altered nucleotide(s) underlined
htxB_W52Y-R	TACGCTGATGGCCGCATAGGACTCGGGCGAAAC	Altered nucleotide(s) underlined
htxB_W52F-F	TTTCGCCCGAGTCCTTTGCGGCCATCAGCGTAG	Altered nucleotide(s) underlined
htxB_W52F-R	TACGCTGATGGCCGCAAAGGACTCGGGCGAAAC	Altered nucleotide(s) underlined
htxB_W52A-F	TTTCGCCCGAGTCCGCGGCGGCCATCAGCGTAG	Altered nucleotide(s) underlined
htxB_W52A-R	TACGCTGATGGCCGCCGCGGACTCGGGCGAAAC	Altered nucleotide(s) underlined

Supplementary Table 4. Primers used in this study.

Name	Description	Source
pET21a(+)	Optional N-terminal T7 and C-terminal His6 tags. IPTG inducible T7 promoter. Ampicillin resistant.	Novagen
pET21a(+)::Te_ptxB	*Trichodesmium erythraeum* IMS101 *ptxB* (minus signal peptide and stop codon) cloned into *Nde*I/*Xho*I sites of pET21a(+) in frame with His6 tag.	This study
pET21a(+)::Te_ptxB_Y208F	Y208F variant of *ptxB* generated by Quikchange using pET21a(+)::Te_ptxB as template.	This study
pET21a(+)::Te_ptxB_Y208A	Y208A variant of *ptxB* generated by Quikchange using pET21a(+)::Te_ptxB as template.	This study
pET21a(+)::Pm_phnD	*Prochlorococcus marinus* MIT9301 *phnD* (minus signal peptide and stop codon) cloned into *Nde*I/*Xho*I sites of pET21a(+) in frame with His6 tag. Codon optimised for *E. coli*.	This study
pET21a(+)::Pm_ptxB	*Prochlorococcus marinus* MIT9301 *ptxB* (minus signal peptide and stop codon) cloned into *Nde*I/*Xho*I sites of pET21a(+) in frame with His6 tag. Codon optimised for *E. coli*.	This study
pET21a(+)::Ps_ptxB	*Pseudomonas stutzeri* WM88 *ptxB* (minus signal peptide and stop codon) cloned into *Nde*I/*Xho*I sites of pET21a(+) in frame with His6 tag. Codon optimised for *E. coli*.	This study
pET21a(+)::htxB	*Pseudomonas stutzeri* WM88 *htxB* (minus signal peptide and stop codon) cloned into *Nde*I/*Xho*I sites of pET21a(+) in frame with His6 tag. Codon optimised for *E. coli*.	This study
pET21a(+)::htxB_W52Y	W52Y variant of *htxB* generated by Quikchange using pET21a(+)::htxB as template.	This study
pET21a(+)::htxB_W52F	W52F variant of *htxB* generated by Quikchange using pET21a(+)::htxB as template.	This study
pET21a(+)::htxB_W52A	W52A variant of *htxB* generated by Quikchange using pET21a(+)::htxB as template.	This study

Supplementary Table 5. Plasmids used in this study.

Supplementary References

1. Martínez, A., Tyson, G.W. & Delong, E.F. Widespread known and novel phosphonate utilization pathways in marine bacteria revealed by functional screening and metagenomic analyses. *Environ. Microbiol*. **12**, 222-238 (2010).

2. McSorley, F.R. et al. PhnY and PhnZ comprise a new oxidative pathway for enzymatic cleavage of a carbon-phosphorus bond. *J. Am. Chem. Soc.* **134**, 8364-8367 (2012).

3. Martínez, A., Osburne, M.S., Sharma, A.K., DeLong, E.F. & Chisholm, S.W. Phosphite utilization by the marine picocyanobacterium Prochlorococcus MIT9301. *Environ. Microbiol.* **14**, 1363-1377 (2012).

4. White, A.K. & Metcalf, W.W. Two C-P lyase operons in Pseudomonas stutzeri and their roles in the oxidation of phosphonates, phosphite, and hypophosphite. *J. Bacteriol*. **186**, 4730-4739 (2004).

5. Vaguine, A.A. Richelle, J. & Wodak S.J. SFCHECK: a unified set of procedure for evaluating the quality of macromolecular structure-factor data and their agreement with atomic model. *Acta. Crystallogr*. *D* **55**, 191-205 (1999).

6. Pettersen, E.F. et al. UCSF Chimera--a visualization system for exploratory research and analysis. *J. Comput. Chem.* **25**, 1605-1612 (2004).

7. Dundas, J. et al. CASTp: computed atlas of surface topography of proteins with structural and topographical mapping of functionally annotated residues. *Nucleic Acids Res*. **34**, 116-W118 (2006).

8. Barker, B.L., Stanley, G.G., & Fronczek, F.R. Private Communication (2014).

9. Belabassi, Y., Gushwa, A.F., Richards, A.F. & Montchamp, J.L. Structural analogues of bioactive phosphonic acids: First crystal structure characterization of phosphonothioic and boranophosphonic acids. *Phosphorus Sulfur* **183**, 2214-2228 (2008).

10. Jones, P.G. & Thonnessen, H. Private Communication (2006).

11. Holt, J. et al. On the Resolution of Secondary Phosphine Oxides via Diastereomeric Complex Formation: The Case of tert-Butylphenylphosphine Oxide. *Synthesis-Stuttgart*, **2009**, 2061-2065 (2009).

12. Shaplov, A.S. et al. Novel phosphonated poly(1,3,4-oxadiazole)s: Synthesis in ionic liquid and characterization. *React. Funct. Polym.* **68**, 208-224 (2008).

13. Harling, S. et al. Calcium-mediated Hydrophosphorylation of Organic Isocyanates with Diphenylphosphane Oxide. *Aust. J. Chem.* **66**, 1264-1273 (2013).

14. Neda, I., Sonnenburg, R., Fischer, A., Jones, P. G. & Schmutzler, R. Preparation of N-phosphorylated nitrogen mustards with the benzodiaza- and oxazaphosphorinone ring systems; Hydrolysis of 2-chloro benzodiazaphosphorinones. *Phosphorus Sulfur* **113**, 287-294 (1996).

15. Gliga, A. et al. New Umpolung Catalysts: Reactivity and Selectivity of Terpenol-Based Lithium Phosphonates in Enantioselective Benzoin-Type Couplings. *Eur. J. Org. Chem.*, **2011**, 256-263 (2011).

16. Rasadkina, E.N., Batalova, T.A., Belskii, V.K. & Nifantev, E.E. 1st example of crystalline complex of hydrophosphoryl compound with phenol. *Zh. Obshch. Khim.* **66**, 1039-1040, (1996).
